# Supplementary material for: Nuclear Membrane Protein SUN5 Is Highly Expressed and Promotes Proliferation and Migration in Colorectal Cancer by Regulating the ERK Pathway
Source: Cancers (Basel). 2022 Oct 31;14(21):5368. doi: 10.3390/cancers14215368 (PMC9654567; doi:10.3390/cancers14215368)

Supplementary Data

Table S1. Primers used for RT-qRCR.

Gene	Sequence
**CD164-F**	ACCCGAACGTGACGACTTTAG
**CD164-R**	CGTGTTCCCCACTTGACAATC
**RAD21-F**	GGATAAGAAGCTAACCAAAGCCC
**RAD21-R**	CTCCCAGTAAGAGATGTCCTGAT
**CKAP2-F**	GCAAGATGCTAACATGCCCAA
**CKAP2-R**	TGGCTTTAGGTATAGTGGCTGA
**CCNA2-F**	GGATGGTAGTTTTGAGTCACCAC
**CCNA2-R**	CACGAGGATAGCTCTCATACTGT
**CDKN1A-F**	TGTCCGTCAGAACCCATGC
**CDKN1A-R**	AAAGTCGAAGTTCCATCGCTC
**EGR1-F**	GGTCAGTGGCCTAGTGAGC
**EGR1-R**	GTGCCGCTGAGTAAATGGGA

Table S2. Association between SUN5 expression and clinic pathological features.

Data		SUN5 expression		P-value
	Patients	low	high	
Total	40	16	24	
**Age(years)**				0.7553
<60	21	7	12	
>60	19	9	12	
**Gender**				0.3391
women	18	10	8	
men	22	8	14	
**Clinical grade**				<0.05
I	7	6	1	
II	8	6	2	
III	15	3	12	
IV	10	1	9	
**Differentiation grade**				<0.05
Poorly differentiated	7	1	6	
Moderately differentiated	31	13	18	
Well differentiated	2	2	0	
**Lymph node metastasis**				<0.001
Yes	22	3	19	
No	18	13	5	

Figure S1. The Western blotting analysis of pERK1/2 level in SUN5-OE LoVo and DLD-1 cells treated with different concentrations of PD0325901 (0.1, 1, 10, 50 and 100 μM) for 48 h. "Ctrl" as the control group, added the solvent (DMSO).

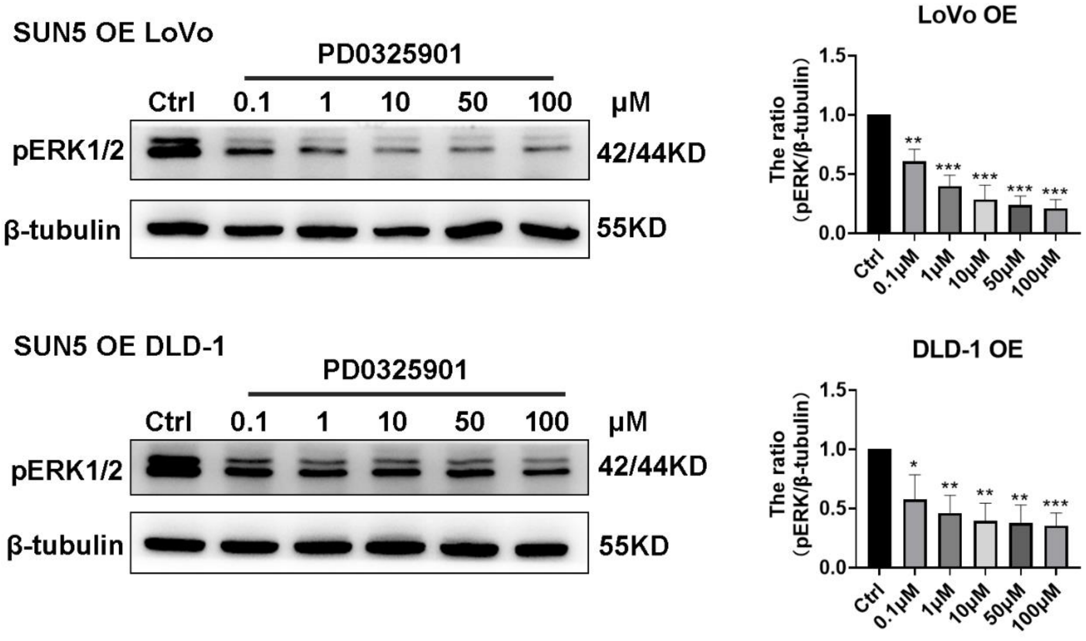

Fig 1:

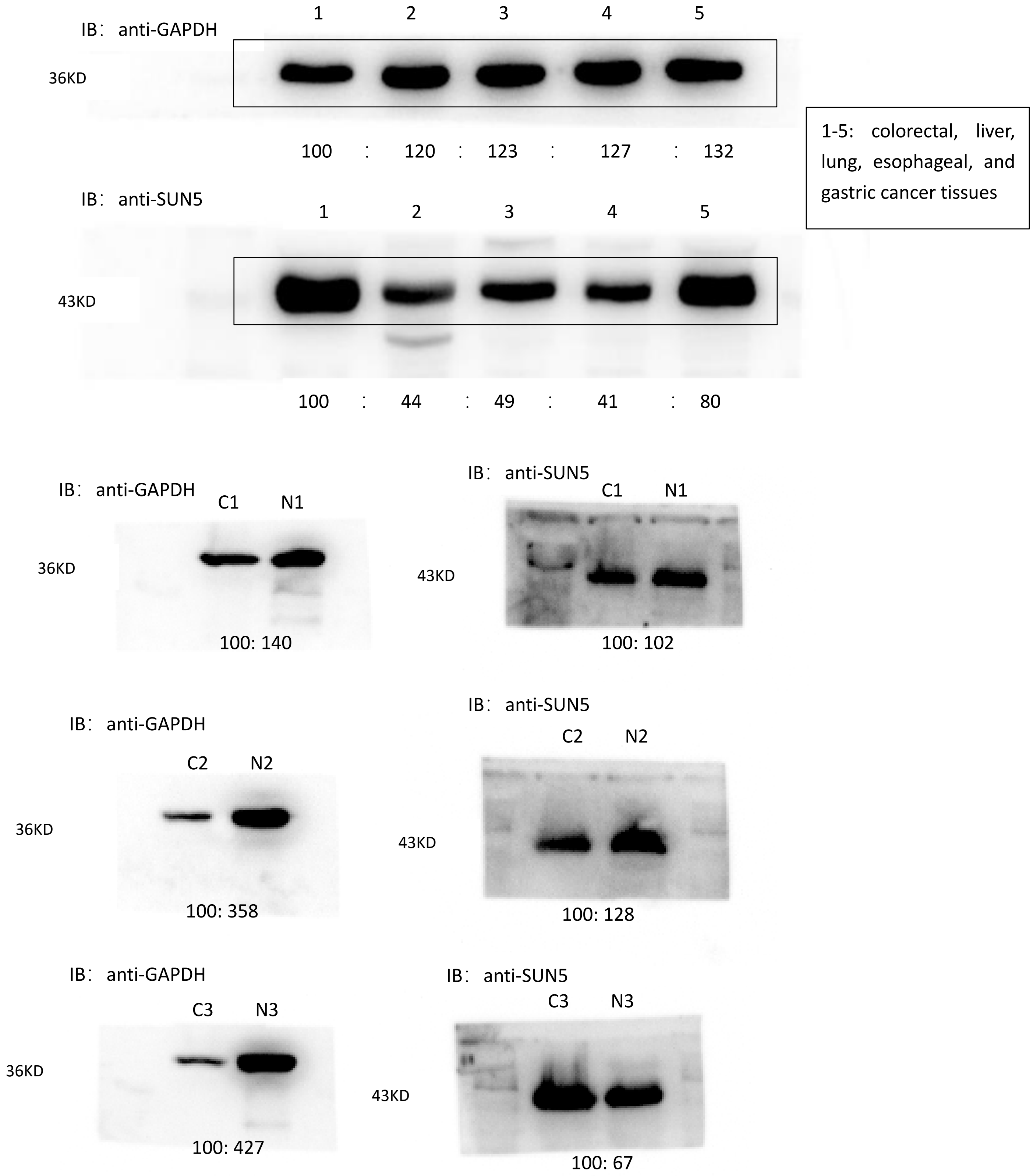
IB: anti-GAPDH
1 2 3 4 5
36KD
100 : 120 : 123 : 127 : 132
IB: anti-SUN5
1 2 3 4 5
43KD
100 : 44 : 49 : 41 : 80
1-5: colorectal, liver, lung, esophageal, and gastric cancer tissues
IB: anti-GAPDH
C1 N1
36KD
100: 140
IB: anti-SUN5
C1 N1
43KD
100: 102
IB: anti-GAPDH
C2 N2
36KD
100: 358
IB: anti-SUN5
C2 N2
43KD
100: 128
IB: anti-GAPDH
C3 N3
36KD
100: 427
IB: anti-SUN5
C3 N3
43KD
100: 67

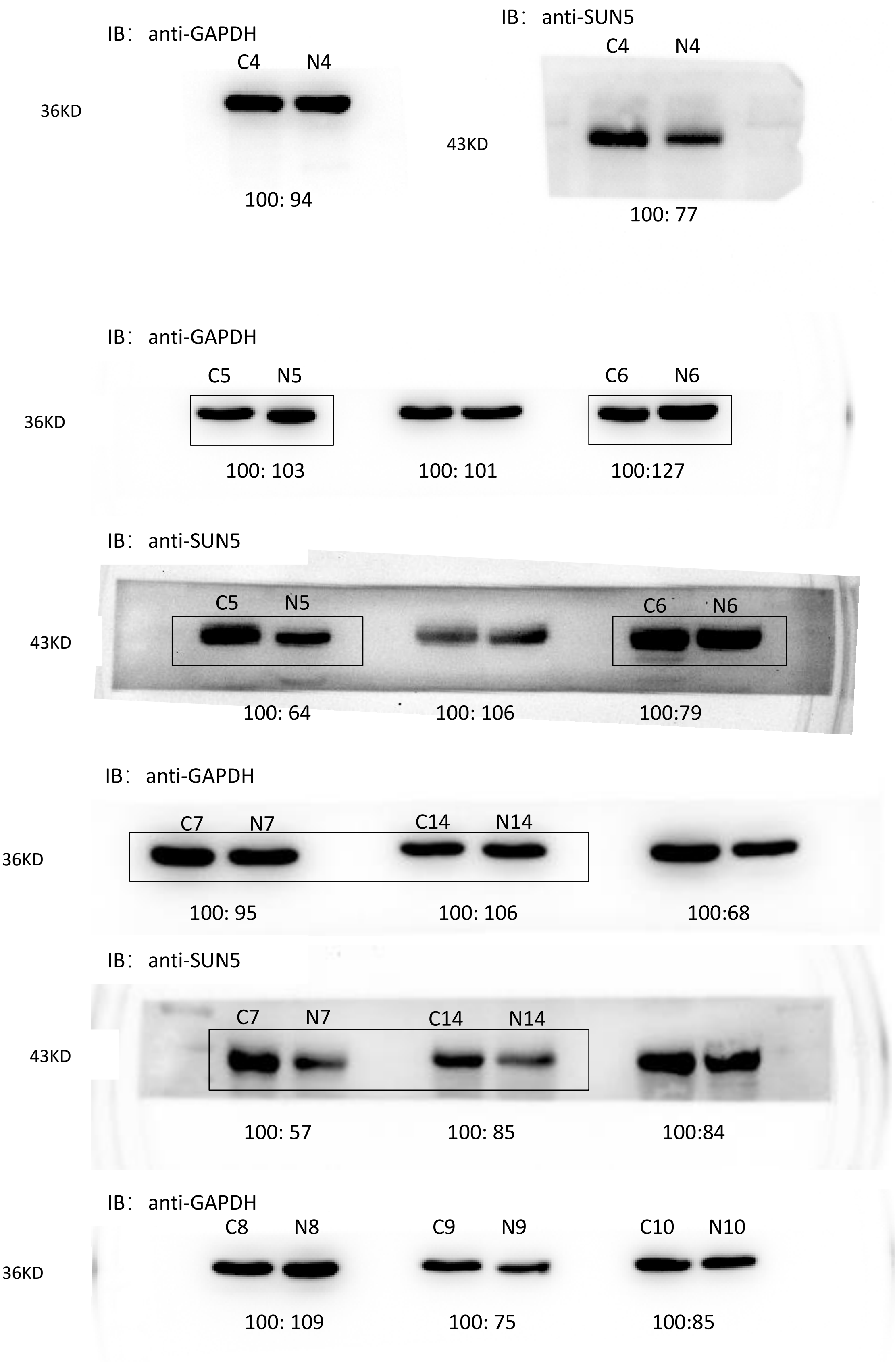
IB: anti-GAPDH
C4 N4
36KD
100: 94
IB: anti-SUN5
C4 N4
43KD
100: 77
IB: anti-GAPDH
C5 N5
C6 N6
36KD
100: 103
100: 101
100:127
IB: anti-SUN5
C5 N5
C6 N6
43KD
100: 64
100: 106
100:79
IB: anti-GAPDH
C7 N7
C14 N14
36KD
100: 95
100: 106
100:68
IB: anti-SUN5
C7 N7
C14 N14
43KD
100: 57
100: 85
100:84
IB: anti-GAPDH
C8 N8
C9 N9
C10 N10
36KD
100: 109
100: 75
100:85

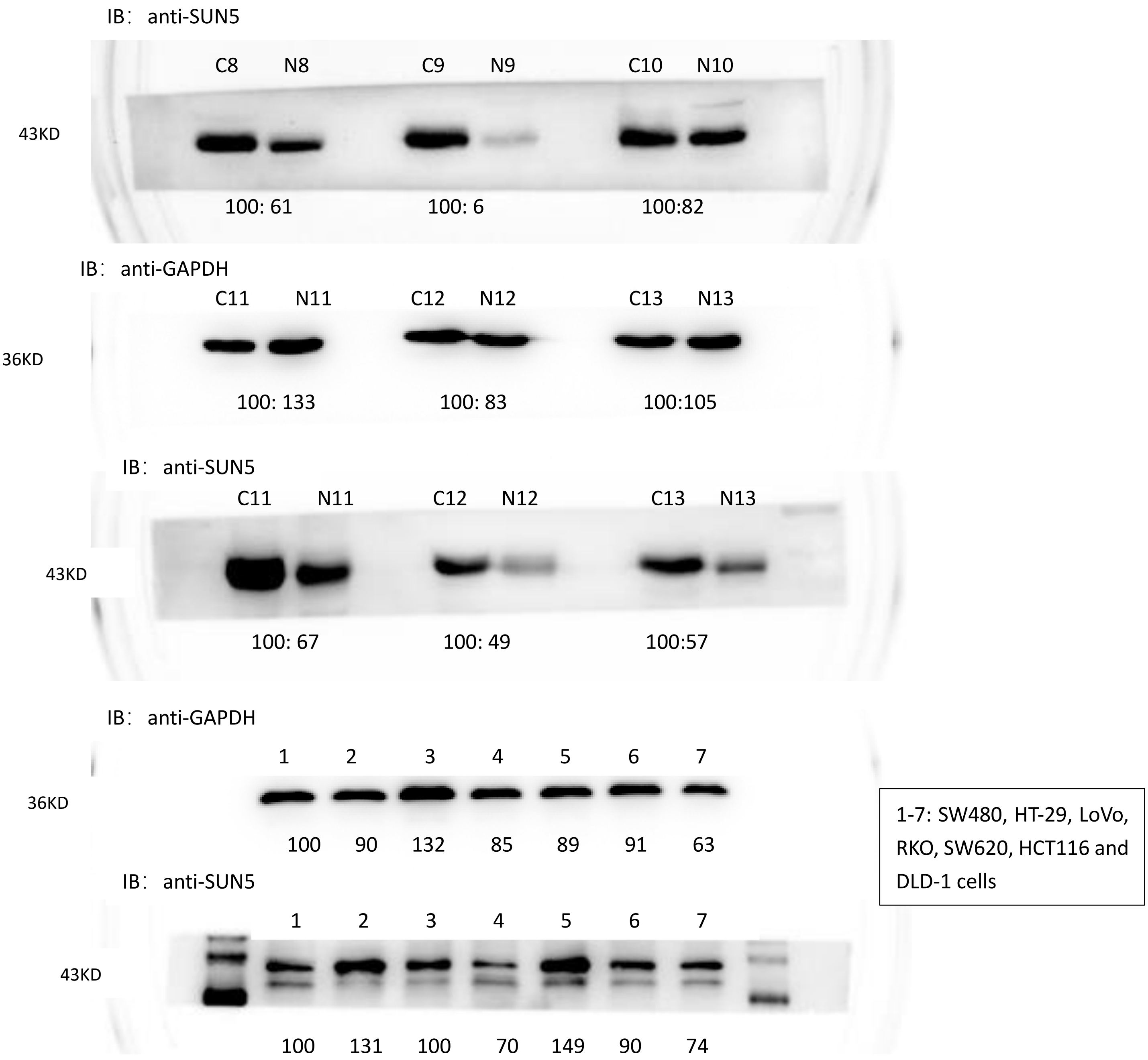
IB: anti-SUN5
C8 N8 C9 N9 C10 N10
43KD
100: 61
100: 6
100:82
IB: anti-GAPDH
C11 N11 C12 N12 C13 N13
36KD
100: 133
100: 83
100:105
IB: anti-SUN5
C11 N11 C12 N12 C13 N13
43KD
100: 67
100: 49
100:57
IB: anti-GAPDH
1 2 3 4 5 6 7
36KD
100 90 132 85 89 91 63
IB: anti-SUN5
1 2 3 4 5 6 7
43KD
100 131 100 70 149 90 74
1-7: SW480, HT-29, LoVo, RKO, SW620, HCT116 and DLD-1 cells

Fig 2：

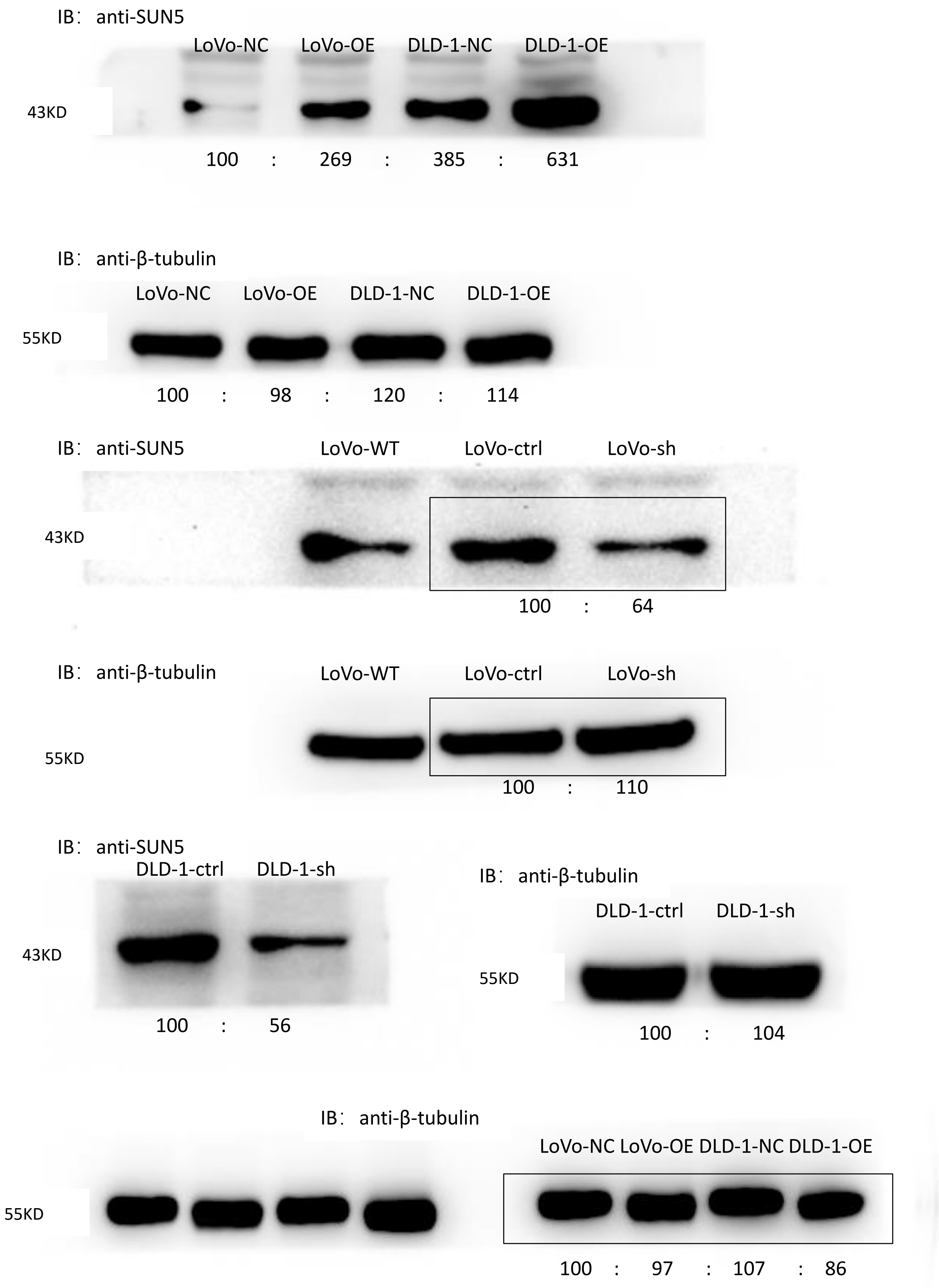
IB：anti-SUN5
LoVo-NC
LoVo-OE
DLD-1-NC
DLD-1-OE
43KD
100 : 269 : 385 : 631
IB：anti-β-tubulin
LoVo-NC
LoVo-OE
DLD-1-NC
DLD-1-OE
55KD
100 : 98 : 120 : 114
IB：anti-SUN5
LoVo-WT
LoVo-ctrl
LoVo-sh
43KD
100 : 64
IB：anti-β-tubulin
LoVo-WT
LoVo-ctrl
LoVo-sh
55KD
100 : 110
IB：anti-SUN5
DLD-1-ctrl
DLD-1-sh
43KD
100 : 56
IB：anti-β-tubulin
DLD-1-ctrl
DLD-1-sh
55KD
100 : 104
IB：anti-β-tubulin
LoVo-NC LoVo-OE DLD-1-NC DLD-1-OE
55KD
100 : 97 : 107 : 86

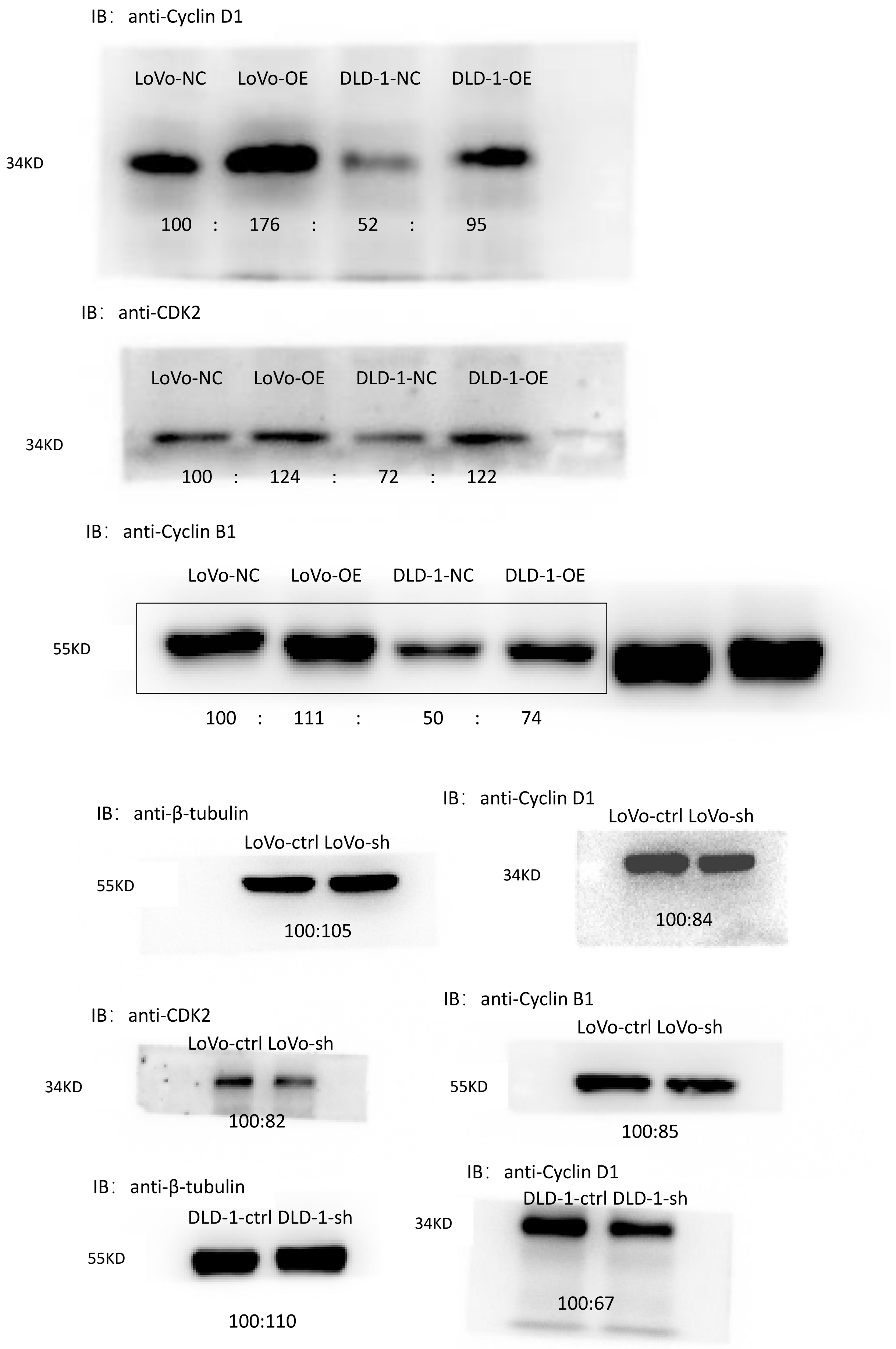
IB： anti-Cyclin D1
LoVo-NC LoVo-OE DLD-1-NC DLD-1-OE
34KD
100 : 176 : 52 : 95
IB： anti-CDK2
LoVo-NC LoVo-OE DLD-1-NC DLD-1-OE
34KD
100 : 124 : 72 : 122
IB： anti-Cyclin B1
LoVo-NC LoVo-OE DLD-1-NC DLD-1-OE
55KD
100 : 111 : 50 : 74
IB： anti-β-tubulin
LoVo-ctrl LoVo-sh
55KD
100:105
IB： anti-Cyclin D1
LoVo-ctrl LoVo-sh
34KD
100:84
IB： anti-CDK2
LoVo-ctrl LoVo-sh
34KD
100:82
IB： anti-Cyclin B1
LoVo-ctrl LoVo-sh
55KD
100:85
IB： anti-β-tubulin
DLD-1-ctrl DLD-1-sh
55KD
100:110
IB： anti-Cyclin D1
DLD-1-ctrl DLD-1-sh
34KD
100:67

IB： anti-CDK2

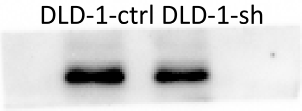

IB： anti-Cyclin B1

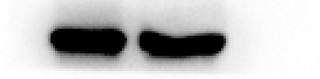

Fig 3：

IB： anti-GAPDH LoVo-NC LoVo-OE DLD-1-NC DLD-1-OE

36KD

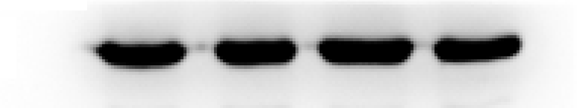

100 : 98 : 117 : 97

IB： anti-E-cadherin LoVo-NC LoVo-OE DLD-1-NC DLD-1-OE

130KD

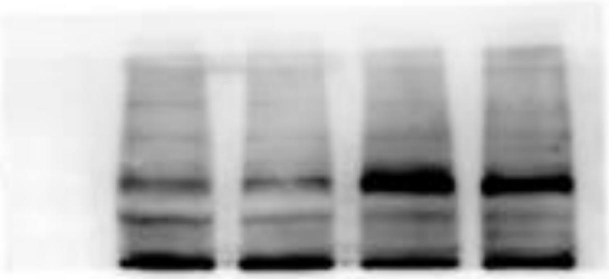

100 : 65 : 351 : 294

IB： anti-N-cadherin LoVo-NC LoVo-OE DLD-1-NC DLD-1-OE

97KD

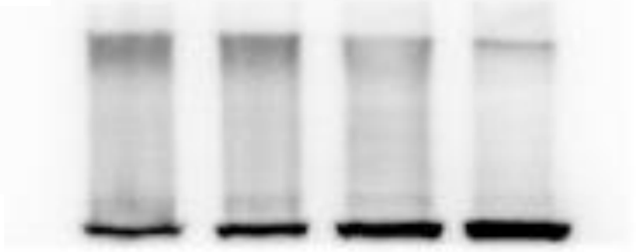

100 : 111 : 166 : 192

IB： anti-Vimentin LoVo-NC LoVo-OE DLD-1-NC DLD-1-OE

57KD

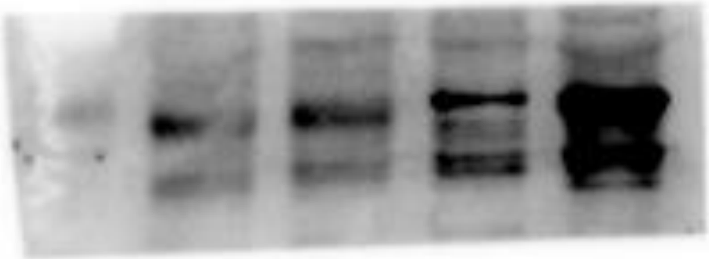

100 : 151 : 148 : 239

IB： anti-β-tubulin LoVo-ctrl LoVo-sh

55KD

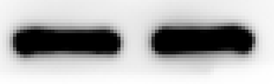

100:103

IB： anti-E-cadherin LoVo-ctrl LoVo-sh

130KD

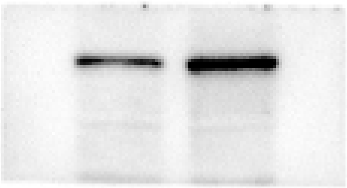

100:192

IB： anti-N-cadherin

LoVo-ctrl LoVo-sh

97KD

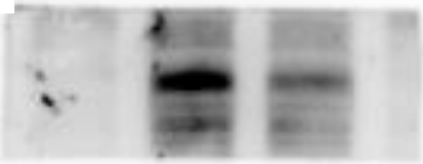

100:73

IB： anti-Vimentin

LoVo-ctrl LoVo-sh

57KD

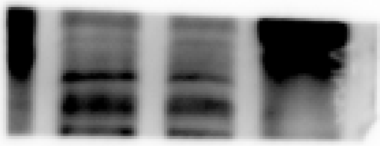

100:76

IB： anti-β-tubulin

DLD-1-ctrl DLD-1-sh

55KD

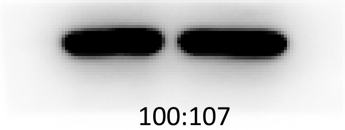

100:107

IB： anti-E-cadherin

DLD-1-ctrl DLD-1-sh

130KD

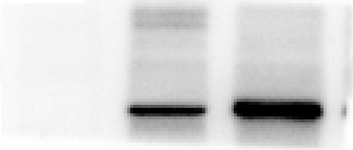

100:170

IB： anti-N-cadherin

DLD-1-ctrl DLD-1-sh

97KD

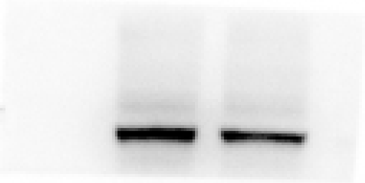

100:81

IB： anti-Vimentin

DLD-1-ctrl DLD-1-sh

57KD

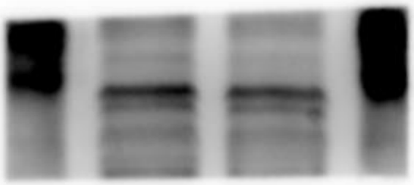

100:88

Fig 4:

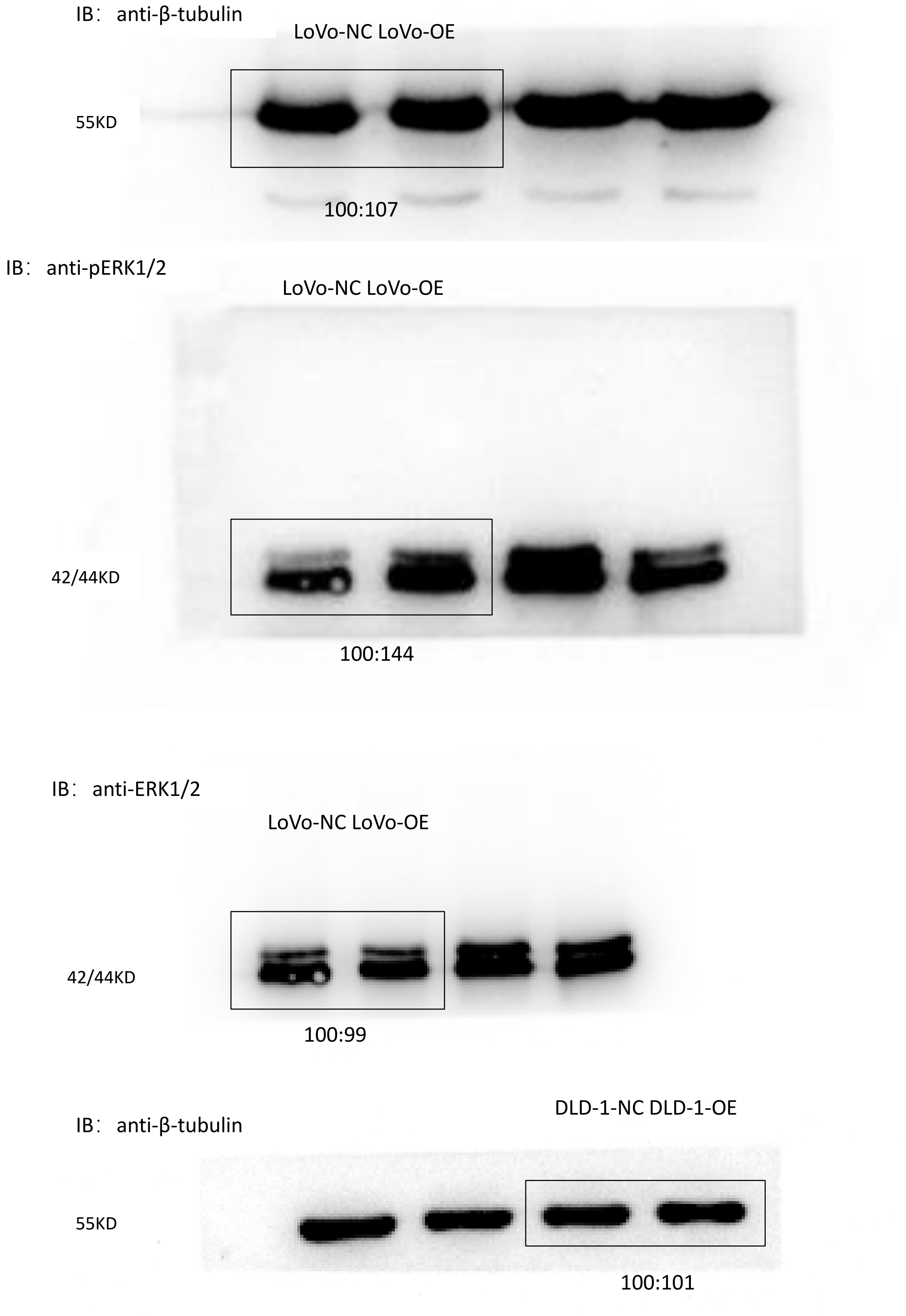

IB: anti-β-tubulin
LoVo-NC LoVo-OE
55KD
100:107
IB: anti-pERK1/2
LoVo-NC LoVo-OE
42/44KD
100:144
IB: anti-ERK1/2
LoVo-NC LoVo-OE
42/44KD
100:99
IB: anti-β-tubulin
DLD-1-NC DLD-1-OE
55KD
100:101

IB：anti-pERK1/2

DLD-1-NC DLD-1-OE

42/44KD

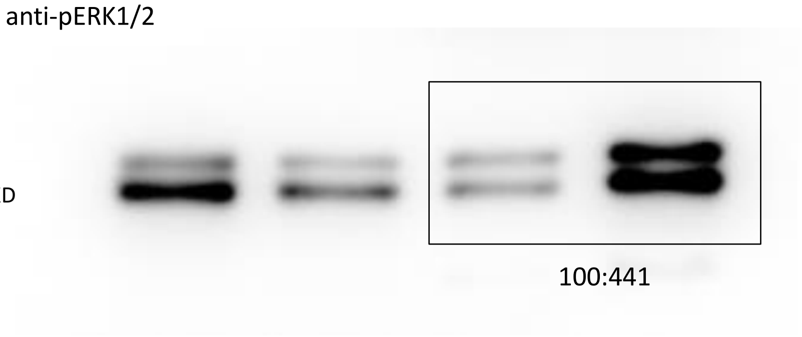

100:441

IB：anti-ERK1/2

DLD-1-NC DLD-1-OE

42/44KD

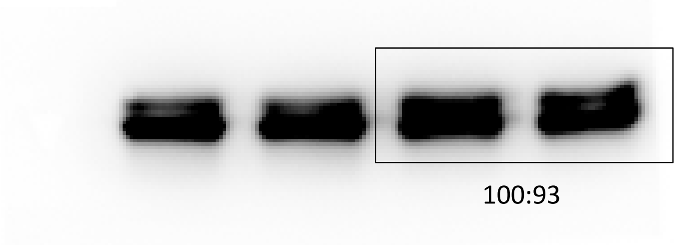

100:93

IB：anti-β-tubulin

LoVo-ctrl LoVo-sh

55KD

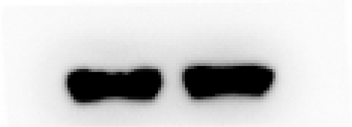

100:97

IB：anti-pERK1/2

LoVo-ctrl LoVo-sh

42/44KD

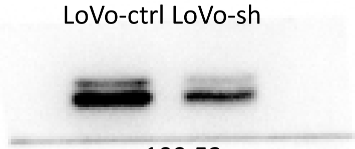

100:53

IB：anti-ERK1/2

LoVo-ctrl LoVo-sh

42/44KD

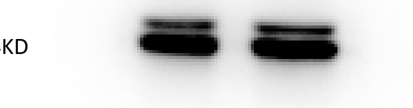

100:109

IB：anti-β-tubulin

DLD-1-ctrl DLD-1-sh

55KD

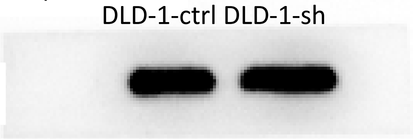

100:112

IB：anti-pERK1/2

DLD-1-ctrl DLD-1-sh

42/44KD

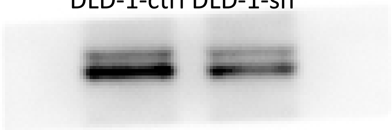

100:59

IB：anti-ERK1/2

DLD-1-ctrl DLD-1-sh

42/44KD

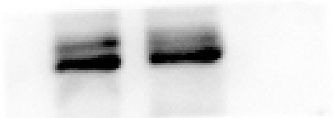

100:97

Fig 5:

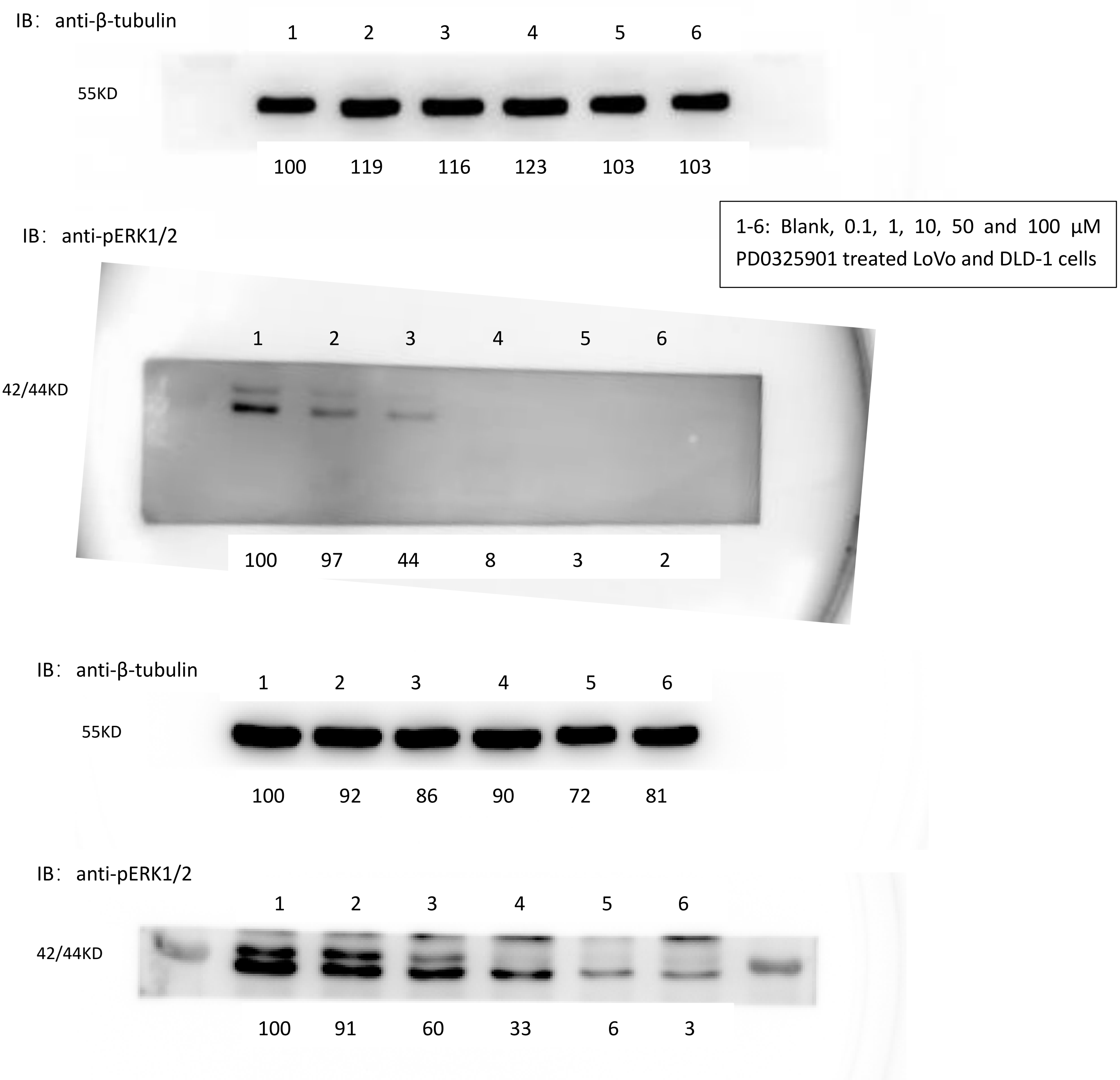
IB：anti-β-tubulin
1 2 3 4 5 6
55KD
100 119 116 123 103 103
1-6: Blank, 0.1, 1, 10, 50 and 100 μM PD0325901 treated LoVo and DLD-1 cells
IB：anti-pERK1/2
1 2 3 4 5 6
42/44KD
100 97 44 8 3 2
IB：anti-β-tubulin
1 2 3 4 5 6
55KD
100 92 86 90 72 81
IB：anti-pERK1/2
1 2 3 4 5 6
42/44KD
100 91 60 33 6 3

Fig 6：

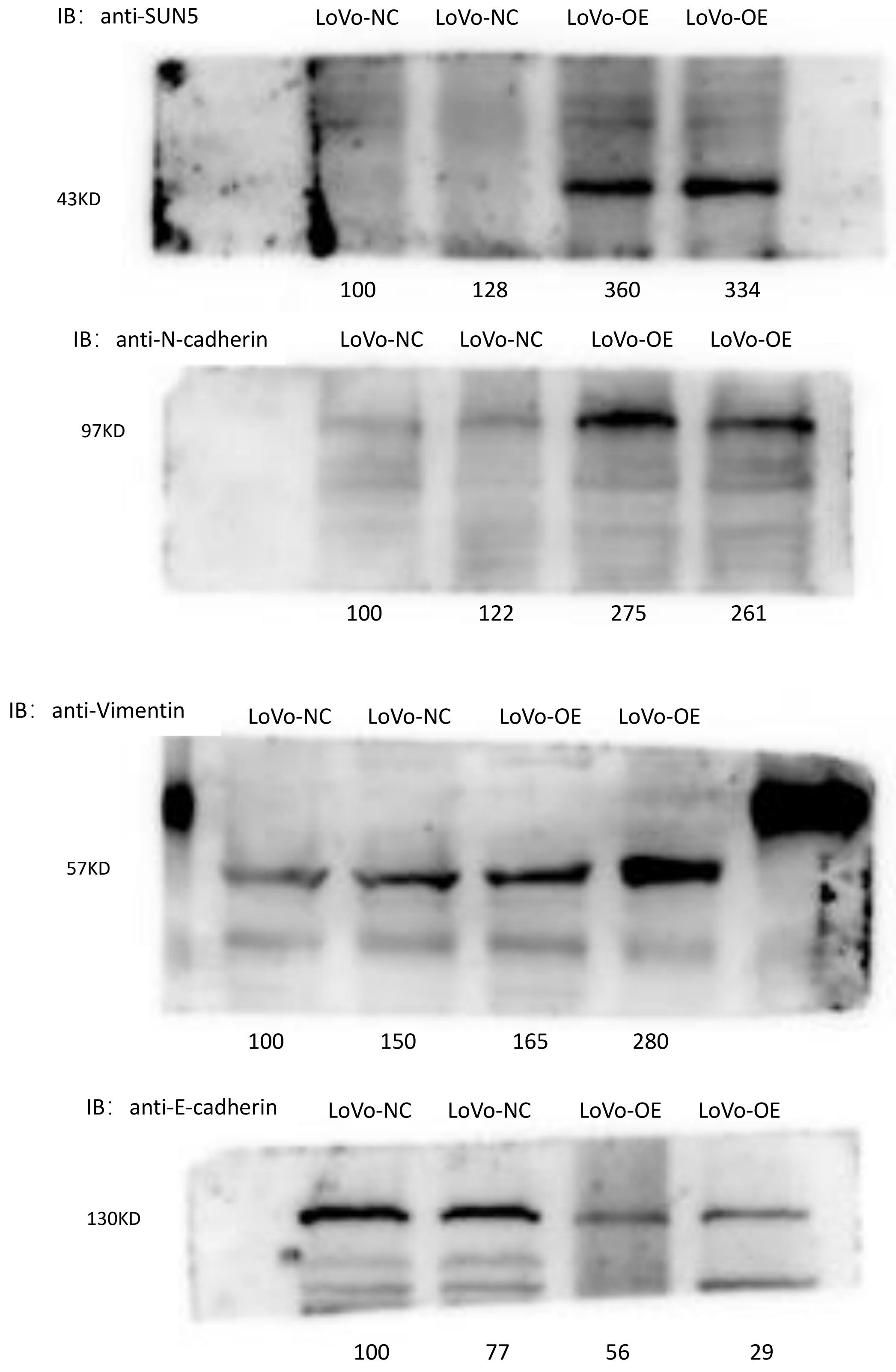
IB：anti-SUN5
LoVo-NC
LoVo-NC
LoVo-OE
LoVo-OE
43KD
100
128
360
334
IB：anti-N-cadherin
LoVo-NC
LoVo-NC
LoVo-OE
LoVo-OE
97KD
100
122
275
261
IB：anti-Vimentin
LoVo-NC
LoVo-NC
LoVo-OE
LoVo-OE
57KD
100
150
165
280
IB：anti-E-cadherin
LoVo-NC
LoVo-NC
LoVo-OE
LoVo-OE
130KD
100
77
56
29

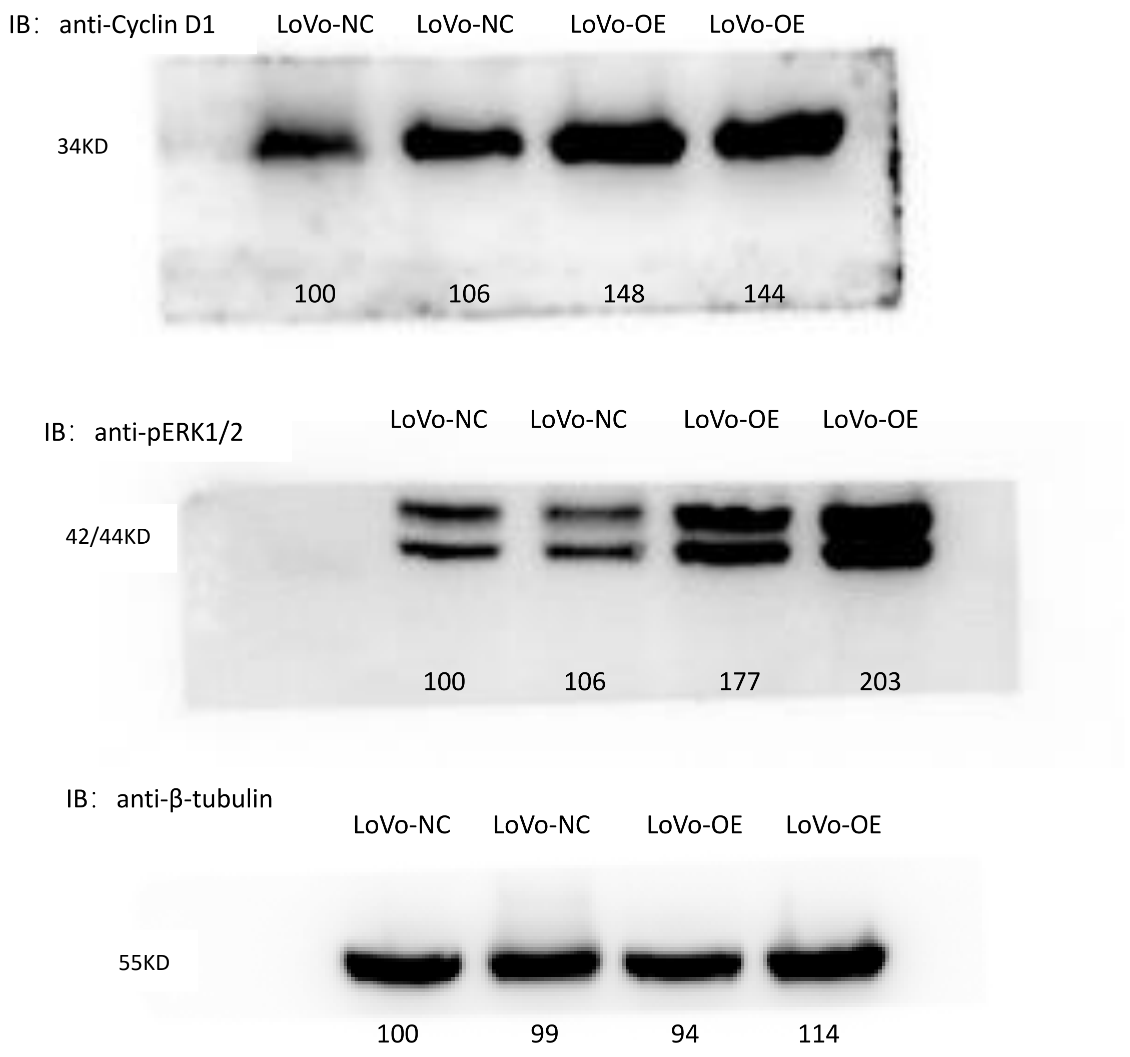

IB：anti-Cyclin D1
LoVo-NC LoVo-NC LoVo-OE LoVo-OE
34KD
100 106 148 144
IB：anti-pERK1/2
LoVo-NC LoVo-NC LoVo-OE LoVo-OE
42/44KD
100 106 177 203
IB：anti-β-tubulin
LoVo-NC LoVo-NC LoVo-OE LoVo-OE
55KD
100 99 94 114

Fig 7:

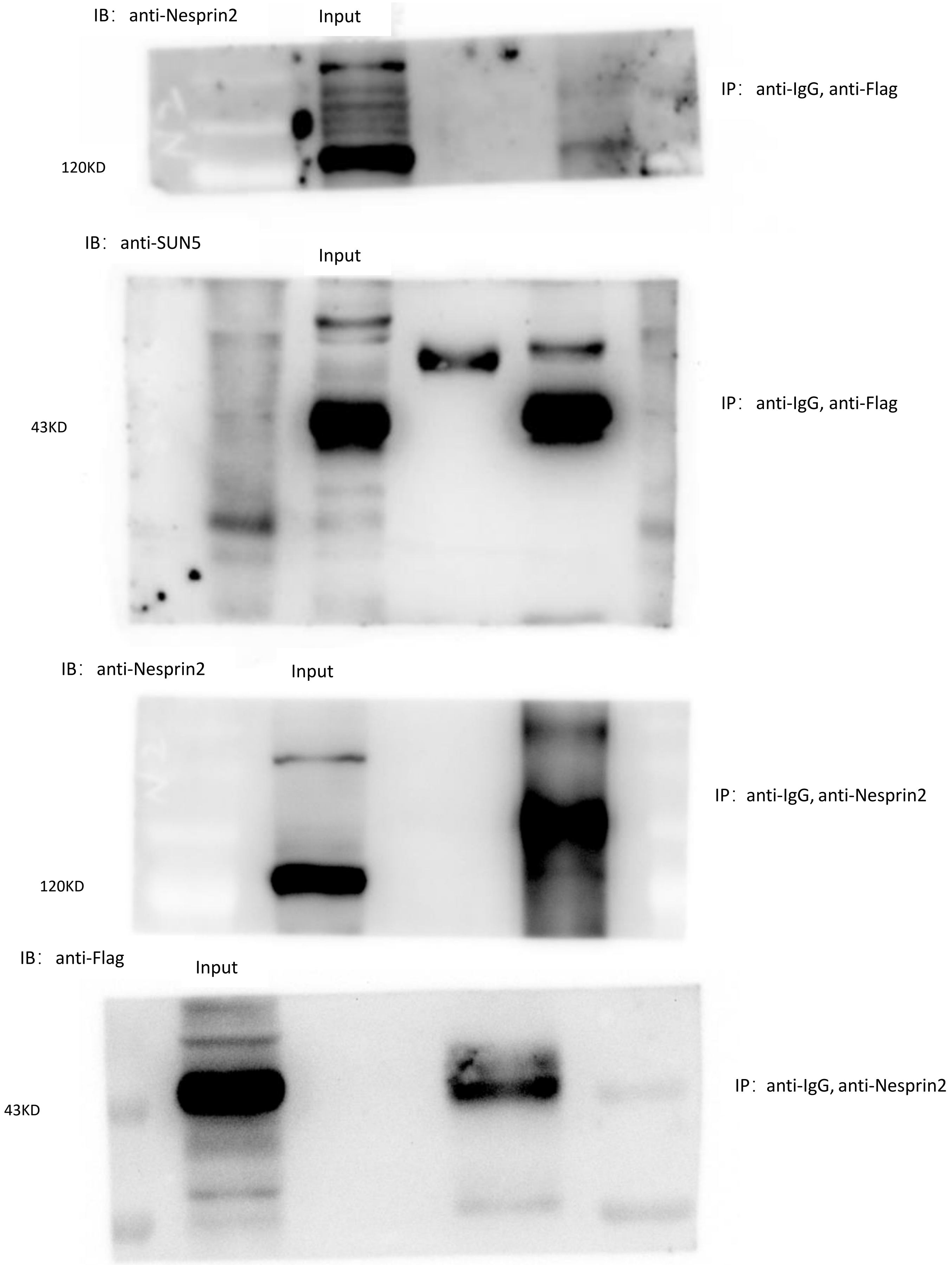
IB: anti-Nesprin2
Input
120KD
IP: anti-IgG, anti-Flag
IB: anti-SUN5
Input
43KD
IP: anti-IgG, anti-Flag
IB: anti-Nesprin2
Input
120KD
IP: anti-IgG, anti-Nesprin2
IB: anti-Flag
Input
43KD
IP: anti-IgG, anti-Nesprin2

IB： anti-Nesprin2 Input

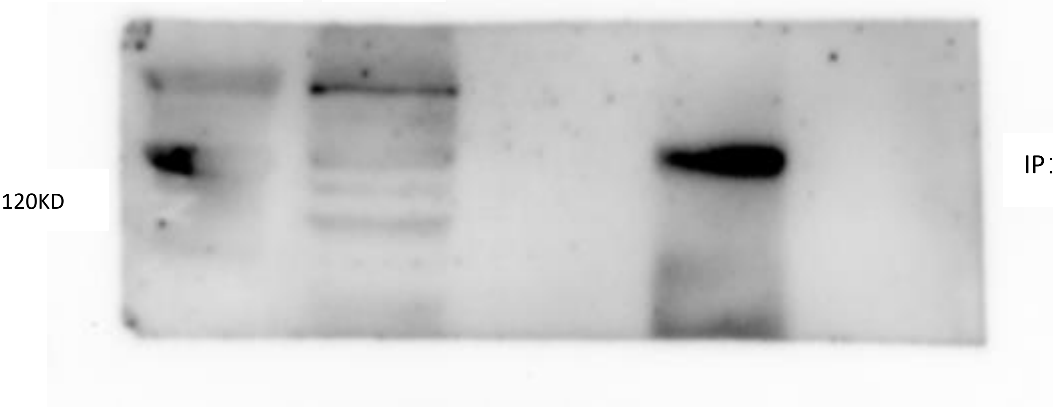

120KD

IP： anti-IgG, anti-ERK1/2

IB： anti-ERK1/2 Input

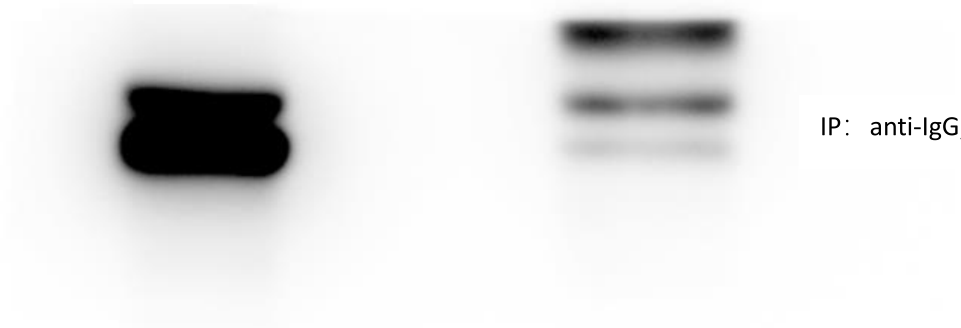

42/44KD

IP： anti-IgG, anti-ERK1/2

IB： anti-Nesprin2 Input

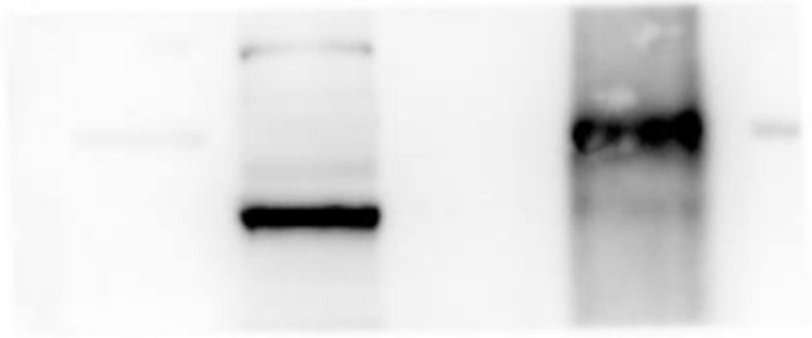

120KD

IP：anti-IgG, anti-Nesprin2

IB： anti-ERK1/2 Input

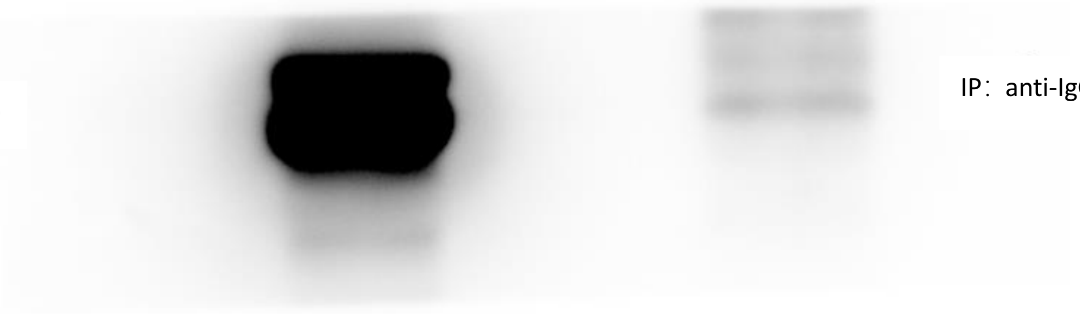

42/44KD

IP：anti-IgG, anti-Nesprin2

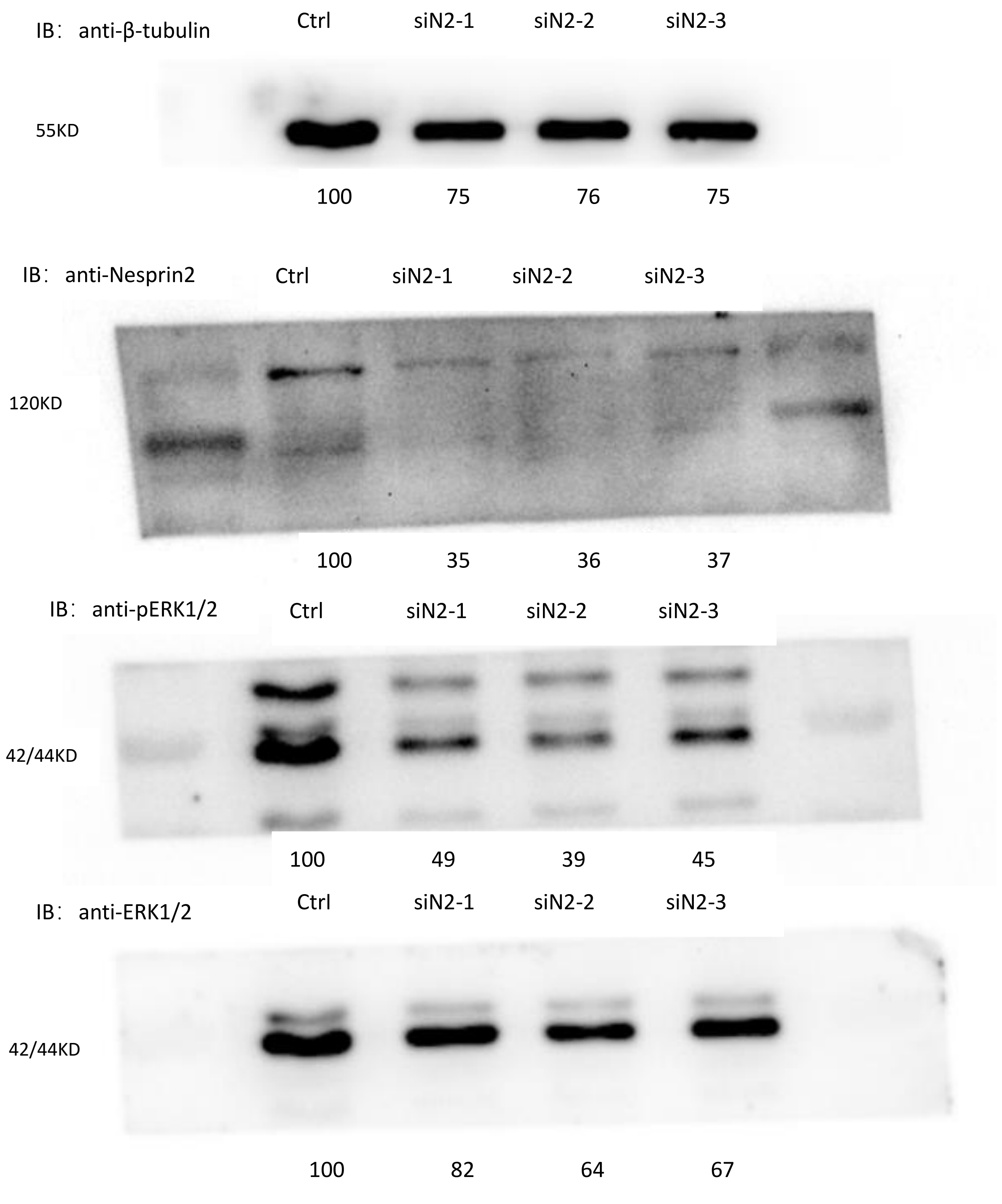
IB：anti-β-tubulin
Ctrl
siN2-1
siN2-2
siN2-3
55KD
100
75
76
75
IB：anti-Nesprin2
Ctrl
siN2-1
siN2-2
siN2-3
120KD
100
35
36
37
IB：anti-pERK1/2
Ctrl
siN2-1
siN2-2
siN2-3
42/44KD
100
49
39
45
IB：anti-ERK1/2
Ctrl
siN2-1
siN2-2
siN2-3
42/44KD
100
82
64
67

Fig 8:

IB: anti-pERK1/2

LoVo-C-NC, LoVo-C-OE, LoVo-N-NC, LoVo-N-OE

42/44KD

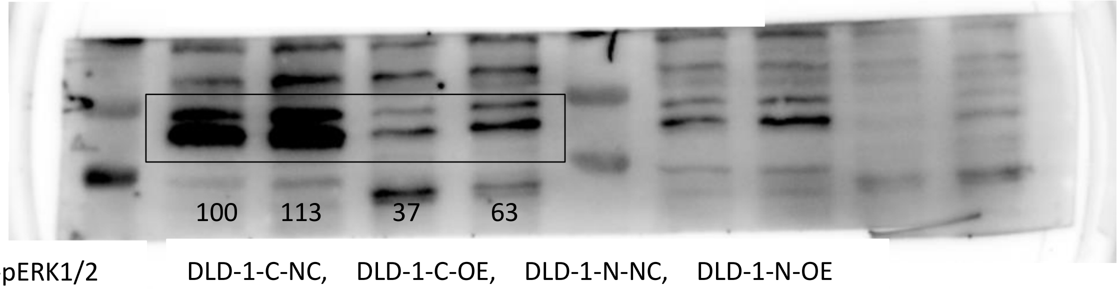

IB: anti-pERK1/2

DLD-1-C-NC, DLD-1-C-OE, DLD-1-N-NC, DLD-1-N-OE

42/44KD

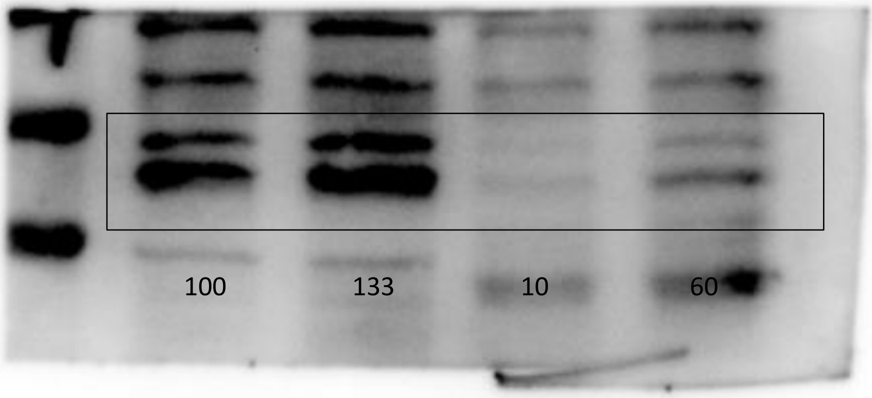

IB: anti-Lamin B1

LoVo-C-NC, LoVo-C-OE, LoVo-N-NC, LoVo-N-OE, DLD-1-C-NC, DLD-1-C-OE, DLD-1-N-NC, DLD-1-N-OE

66KD

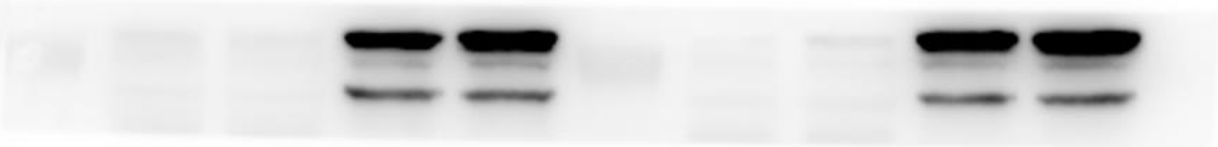

100 125 126 150

IB: anti-GAPDH

LoVo-C-NC, LoVo-C-OE, LoVo-N-NC, LoVo-N-OE, DLD-1-C-NC, DLD-1-C-OE, DLD-1-N-NC, DLD-1-N-OE

36KD

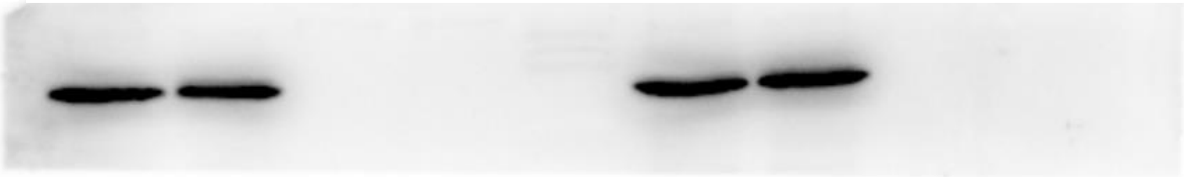

100 87 106 114

IB: anti-Nup93

Input

93KD

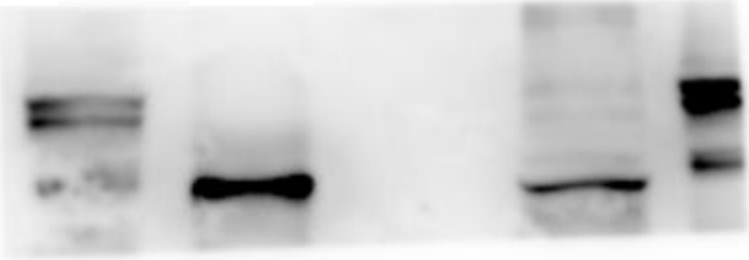

IP: anti-IgG, anti-Flag

IB：anti-SUN5 Input

43KD

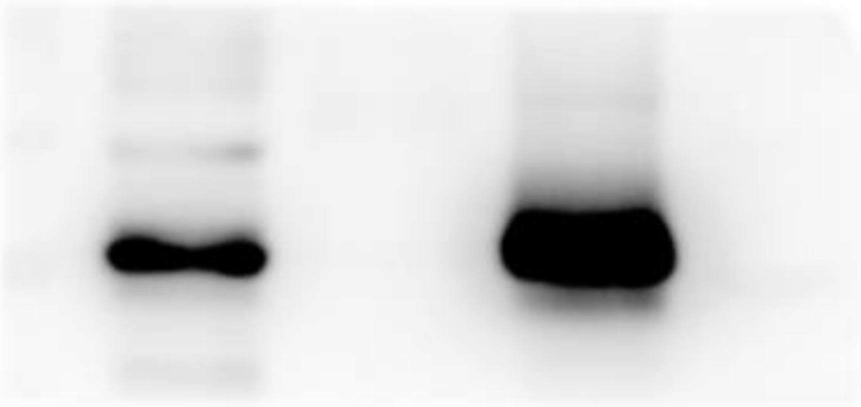

IP：anti-IgG, anti-Flag

IB：anti-Nup93 Input

IP：anti-IgG, anti-Nup93

93KD

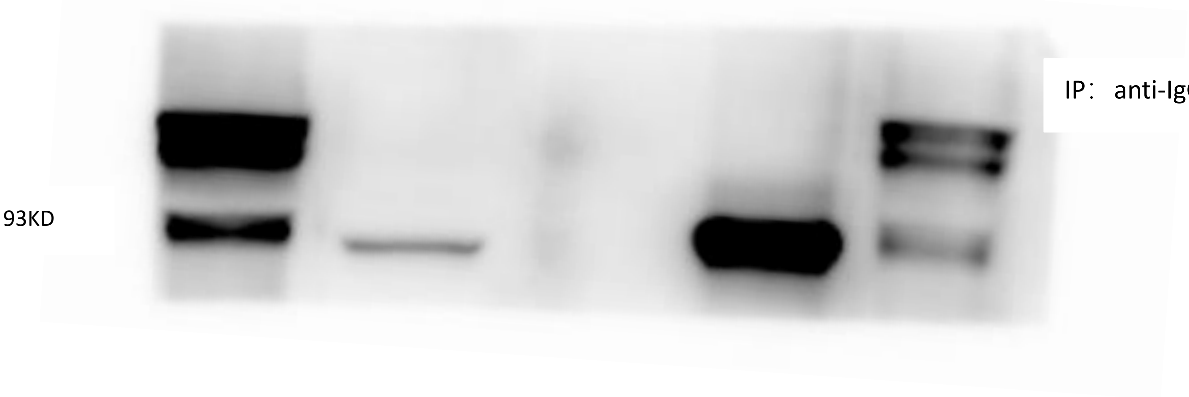

IB：anti-Flag Input

IP：anti-IgG, anti-Nup93

43KD

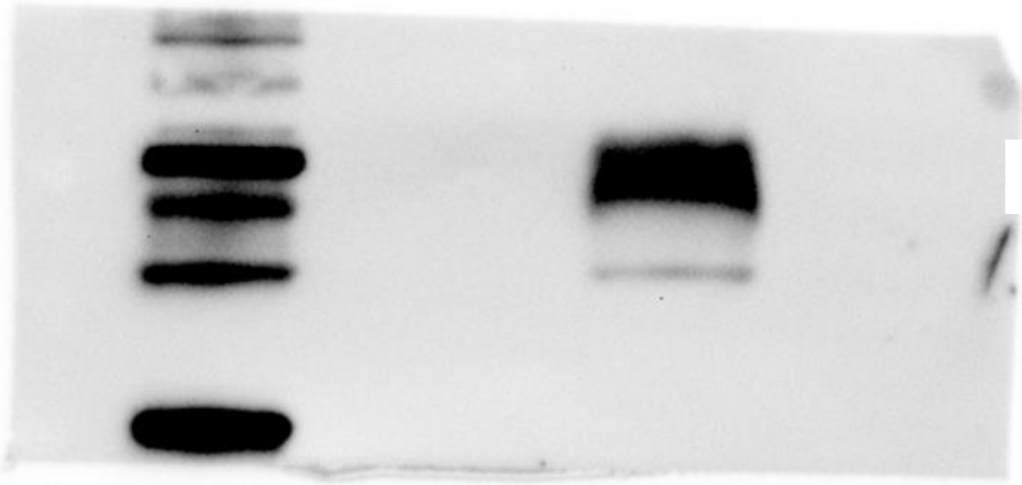

Supplement: Supplementary file 1 [file cancers-14-05368-s001.zip › cancers-1961896-supplementary.pdf]
